# PHYSICAL FUNCTION AND LONGITUDINAL CHANGES IN COGNITIVE FUNCTION

**DOI:** 10.1093/geroni/igz038.3005

**Published:** 2019-11-08

**Authors:** Jinjiao Wang, Jinjiao Wang, Dexia Kong, XinQi Dong

**Affiliations:** 1 University of Rochester, Rochester, New York, United States; 2 University of Rochester School of Nursing, Rochester, New York, United States; 3 Rutgers University, The State University of New Jersey, New Brunswick, New Jersey, United States

## Abstract

Among 2,038 older Chinese adults in the U.S., we examined the relationship between physical function (Short Performance Physical Battery [SPPB], [instrumental] activities of daily living [ADL/IADL] limitations) at baseline (2011-2013) and changes in cognitive function in the two-year follow-up (2013-2015). Cognitive function was measured by the East Boston Memory Test (EBMT), the Digit Span Backwards assessment (DSB), the Symbol Digit Modalities Test (SDMT), and the Mini-Mental State Examination (MMSE). During the two-year follow-up, 41.8%-50.88% of the participants decreased in cognitive function and 32.88%-44.8% increased. In linear regression that adjusted for baseline cognitive function, education, age, and other covariates, baseline SPPB and ADL/IADL limitations were significantly associated with changes in cognitive function in the two-year follow-up (SPPB: βEBMT=0.0149, p<0.05; βDSB=0.0253, p>0.05; βSDMT=0.2742, p<0.01; βMMSE=0.1070, p<0.001; ADL/IADL limitations: βEBMT= -0.0401, p<0.0001; βDSB= -0.0410, p<0.05; βSDMT= -0.3027, p<0.01; βMMSE= -0.2566, p<0.0001). This suggests that better physical function predicts positive changes in cognitive function.

